# Genome-Wide Sequencing of Cellular microRNAs Identifies a Combinatorial Expression Signature Diagnostic of Sepsis

**DOI:** 10.1371/journal.pone.0075918

**Published:** 2013-10-16

**Authors:** Yuqian Ma, David Vilanova, Kerem Atalar, Olivier Delfour, Jonathan Edgeworth, Marlies Ostermann, Maria Hernandez-Fuentes, Sandrine Razafimahatratra, Bernard Michot, David H. Persing, Ingrid Ziegler, Bianca Törös, Paula Mölling, Per Olcén, Richard Beale, Graham M. Lord

**Affiliations:** 1 Department of Experimental Immunobiology, King's College London, London, United Kingdom; 2 Division of Asthma, Allergy and Lung Biology, King's College London, London, United Kingdom; 3 NIHR Biomedical Research Centre, Guy's and St Thomas' NHS Foundation Trust and King's College London, London, United Kingdom; 4 R&D, Cepheid Europe, Maurens Scopont, France; 5 Guy's and St Thomas NHS Foundation Trust, London, United Kingdom; 6 Cepheid, Sunnyvale, California, United States of America; 7 Department of Infectious Diseases, Örebro University Hospital, Örebro, Sweden; 8 Department of Laboratory Medicine, Clinical Microbiology, Örebro University Hospital, Örebro, Sweden; Cardiff University School of Medicine, United Kingdom

## Abstract

**Rationale:**

Sepsis is a common cause of death in the intensive care unit with mortality up to 70% when accompanied by multiple organ dysfunction. Rapid diagnosis and the institution of appropriate antibiotic therapy and pressor support are therefore critical for survival. MicroRNAs are small non-coding RNAs that play an important role in the regulation of numerous cellular processes, including inflammation and immunity.

**Objectives:**

We hypothesized changes in expression of microRNAs during sepsis may be of diagnostic value in the intensive care unit (ICU).

**Methods:**

Massively parallel sequencing of microRNAs was utilised for screening microRNA candidates. Putative microRNAs were validated using quantitative real-time PCR (qRT-PCR). This study includes data from both a training cohort (UK) and an independent validation cohort (Sweden). A linear discriminant statistical model was employed to construct a diagnostic microRNA signature.

**Results:**

A panel of known and novel microRNAs were detectable in the blood of patients with sepsis. After qRT-PCR validation, microRNA miR-150 and miR-4772-5p-iso were able to discriminate between patients who have systemic inflammatory response syndrome and patients with sepsis. This finding was also validated in independent cohort with an average diagnostic accuracy of 86%. Fractionating the cellular components of blood reveals miR-4772-5p-iso is expressed differentially in monocytes. Functional experiments using primary human monocytes demonstrate that it expressed in response to TLR ligation.

**Conclusions:**

Taken together, these data provide a novel microRNA signature of sepsis that should allow rapid point-of-care diagnostic assessment of patients on ICU and also provide greater insight into the pathobiology of this severe disease.

## Introduction

Sepsis and its sequelae constitute a major health problem in the developed world in terms of morbidity, mortality and cost. An ageing population, higher frequencies of invasive procedures, greater prevalence of multi-drug resistant organisms in hospitals and iatrogenic immunosuppression have led to an expanding population of susceptible individuals. The overall cost of treatment exceeds $3.5 billion per year in the UK and $16.7 billion per year per year in the US [1]. From the perspective of individualized treatment strategies, difficulties in diagnosing sepsis rapidly and accurately have helped contribute to delays in the administration of adequate antibiotic treatment and ICU services. The absence of a validated diagnostic test leads to the empirical use of broad-spectrum antibiotics and the inappropriate deployment of expensive, potentially life-saving technology, without significant improvements in clinical outcomes for affected patients [2], [3]. While tests like C-reactive protein (CRP), procalcitonin, and neutrophil CD64 expression have some value, none possess the essential characteristics required to have a significant impact on morbidity, mortality and cost.

In the US and the EU combined, approximately 1.5 million hospitalized patients are diagnosed with sepsis per year. In the ICU, the most expensive form of care given to hospitalized patients, approximately 15% of patients develop severe sepsis and septic shock [1], [4]. Overall mortality exceeds 40%, and this represents close to 30% of all hospital-based deaths [5]. These data show that there is an unmet healthcare need for a biomarker that could decrease overall mortality, morbidity and healthcare-associated costs as a result of more rapid and accurate diagnosis.

MicroRNAs are a class of RNA molecules that control post-transcriptional gene expression primarily by complementary base pairing with specific “seed” sequences in the 3′UTR of their target mRNAs [6], [7]. The expression levels of specific microRNAs can be of diagnostic value in various forms of malignancy [8], [9], [10] and may also provide insight into disease pathogenesis [9], [11]. Recently, a number of studies have looked at a limited set of microRNAs and have shown that their expression is altered in the context of inflammation or sepsis, both *in vitro* [12], [13] and *in vivo* [10], [14].

Given that the total number of microRNAs in the human genome is incompletely defined and that pre-clinical models of sepsis are not as informative as in other diseases [15], [16], [17], we took the approach of massive parallel sequencing of all microRNAs (mir-seq) present in the circulating blood leucocytes in patients with SIRS, sepsis and healthy controls. Novel microRNA signatures that discriminated between these three clinically-assigned diagnostic categories were then validated by real-time PCR analyses in an observational clinical study. This signature was then cross-validated using samples from an independent cohort. Mechanistic pathways were tested by fractionation of the circulating blood populations and dissection of candidate upstream pathways capable of inducing the expression of these candidate microRNAs *in vitro* in primary human cells.

## Methods

### Ethics Statement

On behalf of all authors, I certify that this study involving human subjects is in accordance with the Helsinki declaration of 1975 as revised in 2008. Study in UK was approved by St Thomas' Hospital Research Ethics Committee/South East London REC 2; and study in Sweden cohort was approved by the regional ethical review board in Uppsala, Sweden. All patients were written informed consent.

### Patients and study design

A clinical study was carried out in the Intensive Care Unit (ICU) at Guy's and St.Thomas' Hospital, London (Ethic approval REC reference No. 08/H0802/110) as training cohort. Eligible patients or healthy volunteers signed informed consent where possible, for patients who were unconscious a signed informed consent was taken by their legal representatives as approved by local ethic committee. For each individual, 20 ml of blood was taken by venepuncture and blood from ICU patients was obtained from existing central venous catheters using EDTA anti-coagulated Vacutainers (BD Biosciences, NJ, USA). Whole blood was stored at 4°C before transfer to the research lab for processing. All studies were carried out in a double-blind fashion with research nurses taking samples and collecting clinical information; laboratory results were generated without knowing the nature of samples. ICU physicians and microbiologists then reviewed the clinical and lab data and grouped the patients according to validated clinical definitions [18] (Table 1). Inclusion and Exclusion Criteria are listed in supplementary data (See Supporting Information S1 in File S1). A total of 23 sepsis patients and 22 SIRS patients were included in this study, together with 21 healthy volunteers.

**Table 1:** Diagnostic criteria for sepsis [18].

**Infection, documented or suspected, and some of the following:**
General variables
Fever (core temperature >38.3°C)
Hypothermia (core temperature <36°C)
Heart rate >90 min^−1^ or >2 SD above the normal value for age
Tachypnea
Altered mental status Significant edema or positive fluid balance (>20 mL/kg over 24 hrs)
Hyperglycemia (plasma glucose >120 mg/dL or >7.7 mmol/L) in the absence of diabetes
Inflammatory variables Leukocytosis (WBC count >12,000 µL^−1^)
Leukopenia (WBC count <4000 µL^−1^)
Normal WBC count with >10% immature forms
Plasma C-reactive protein >2 SD above the normal value
Plasma procalcitonin >2 SD above the normal value
**Hemodynamic variables**
Arterial hypotension (SBP<90 mm Hg, MAP<70, or an SBP decrease >40 mm Hg in adults or <2 SD below normal for age)
SvO2 <70%b; Cardiac index <3.5 L·min^−1^·M^−23^
**Organ dysfunction variables**
Arterial hypoxemia (Pa_O2_/F_IO2_ <300)
Acute oliguria (urine output <0.5 mL·kg^−1^· hr^−1^ or 45 mmol/L for at least 2 hrs); Creatinine increase >0.5 mg/dL
Coagulation abnormalities (INR>1.5 or aPTT>60 secs)
Ileus (absent bowel sounds)
Thrombocytopenia (platelet count <100,000 µL^−1^)
Hyperbilirubinemia (plasma total bilirubin >4 mg/dL or 70 mmol/L)
**Tissue perfusion variables**
Hyperlactatemia (>1 mmol/L)
Decreased capillary refill or mottling

For the validation cohort in Sweden, the study was carried out at The Department of Infectious Diseases, Örebro University Hospital, Sweden (approved by local ethics committee). A total of 1093 patients were recruited from this cohort as previously described [19] and all patients signed informed consent. A positive blood culture was found in 138 patients. A retrospective chart review was performed by a specialist of infectious diseases. 17 patients with positive blood culture and were diagnosed with severe sepsis or septic shock. Another 6 patients had blood culture negative however were diagnosed with clinical infection and severe sepsis or septic shock. Together those 23 patients form the severe sepsis-group of Örebro. 15 patients had negative blood cultures and negative SeptiFast PCR results form the non-infected group of Örebro.

### Small RNA library and HiSeq Sequencing

RNA extraction was performed as described using a standard TRIzol LS protocol [20]. Small RNA fraction purification and sequencing (FASTERIS SA, Switzerland) were performed as previously described [21] and miRbase version 16 was used in this study. cDNA were purified on a gel and the library was quantified before dilution to 10 nM. The diluted cDNA library was then sequenced with a spike PhiX reference on a HiSeq (Illumina) according to the manufacturer's instructions. After adapter removal, reads were mapped to the human genome (NCBI build 37) and only reads having at least 17 nucleotides with an identity of 100% on the human genome are conserved. Reads mapped to miRNAs were counted and normalized by the total number of reads mapping the human genome per million (RPM).

### Quantitative reverse transcription polymerase chain reaction (qRT-PCR) for miRNA Expression

MiRNA levels were detected by qRT-PCR using the Exiqon custom LNA™ primers (Exiqon, Vedbaek, Denmark) according to the manufacturer's instructions. Given the lack of consensus about the validity and accuracy of normalization for microRNAs in biomarker studies, raw Ct values were used [22]. The fold change of each miRNA was calculated from the equation 2^−ΔCT^, where ΔCT = Mean Ct_miRNA-A_−Mean Ct_miRNA-B_ (where Ct is the threshold cycle for a sample). The relative abundance of each miRNA was calculated as the ratio of the value from sepsis to the value from controls (stimulated monocytes to non-stimulated monocytes in *in vitro* experiments), producing a fold change value.

### Peripheral monocyte purification and in vitro stimulation

PBMCs were purified using Ficoll-Hypaque separation method as previously described [23]. Monocytes were positively selected using magnetic beads conjugated to an anti-CD14 antibody (Miltenyi, Germany). Monocytes isolated from healthy donors were subsequently cultured with RPMI-1640 supplemented with 10% FCS at concentration of 1×10^6^/ml in a 6-well culture dish in 5 ml/well. TLR ligands were added into culture for for 24 hrs in 37°C, with 5% CO_2_ at the concentrations suggested by Taganov *et al* [24]: 10 µg/ml peptidoglycan (PGN), 100 ng/ml Pam3CSK4, 25 µg/ml poly(I∶C), 100 ng/ml LPS (*E. coli* 055:B5), 10 µg/ml ultrapure LPS (*E. coli* strain K12), 100 ng/ml recombinant flagellin (*S. typhimurium*), 5 µg/ml imiquimod-R837 and 5 µM CpG oligonucleotide type C (all TLR ligands were purchased from InvivoGen, USA). RNA was harvested at the end of culture using Trizol RNA isolation method for qRT-PCR. In some experiments, patient samples were isolated for both CD14+ monocytes and CD14+ depleted PBMCs using same method, in order to have total RNA for sequencing.

### Statistical Analysis

For sequencing data, raw reads obtained from each library were normalized to RPM. For qRT-PCR, the primary analysis was the comparison between Sepsis and non-sepsis groups (SIRS and healthy subjects). Variables for sepsis prediction were assessed using univariate analysis on the first cohort, and those that were statistically significant (*p*<0.05) in this analysis were included in multivariate analysis by adopting a multiple stepwise linear discriminant analysis (LDA) model based on a p value of <0.05. The second cohort was then used to validate this LDA model. Microsoft Excel 2007, SPSS 17.0 and Graphpad Prism 4.0 were chosen for constructing graphs and statistical analysis. Specific statistical test methods are indicated in the figure legends.

## Results

### Study overview of training and validation cohorts

22 SIRS patients, 23 septic patients and 21 healthy donors were recruited from ICU at Guy's and St.Thomas' Hospital, UK. Within the sepsis group, 7 had Gram negative sepsis (2 bacteremia), 3 had Gram positive sepsis, 1 had a mixture of both and the remaining 11 cases were diagnosed clinically according to validated clinical diagnostic criteria in a blinded manner (Table 2 and Table 3).

**Table 2:** Demographic and clinical information (UK cohort).

	Healthy subjects (n = 21)	Patients with SIRS (n = 22)	Patients with Sepsis (n = 23)	*p*-value between patients group
**Age**	42 (27–61)	57(27–92)	61 (35–87)	0.4008
**Male**	7 (33.3%)	16 (72.7%)	8 (34.9%)	0.0108
**APACHEII score**	—	12 (4–20)	18 (9–27)	0.016
**SOFA score**	—	3 (1–8)	6 (1–14)	0.0319
**1-month mortality rate**	—	7 (31.8%)	0	—
**CRP**	—	53 (5–123)	242 (45–489)	*p*<0.0001
**WBC count**	—	14.8 (12.1–47.0)	16.8 (13.3–28.7)	0.0266
**Neutrophils count**	—	12.55 (6.6–30.1)	14.55 (8.6–26.9)	0.0573
**Temperature**	—	36.8 (34.5–37.9)	36.15 (34.4–38.1)	0.1889

Data are presented as numbers (percentages) for categorical variables and as median values (ranges) for continuous variables. Statistics: The distribution of sex was tested using Pearson Chi-Square Tests, the rest variables were tested using Mann-Whitney U test.

**Table 3:** Clinical diagnosis and microbiological information.

Cohort	Group	Clinical Diagnosis	Microbiological culture results
**UK**	**SIRS**	Coronary Artery Bypass Grafts (n = 9); Aortic dissection (n = 2); Aortic Valve Replacement (n = 1); Trans Apical Aortic Valve Replacement (n = 1); Burn (n = 1); Abdominal Aortic Aneurysm (n = 1); Post operative trauma (n = 4); Others (n = 3)	No relevant positive culture results
	**Sepsis**	Intra-abdominal Sepsis (n = 5); Pneumonia (n = 4); Lower respiratory tract infection (n = 3); Bloodstream infection (n = 3); Biliary Sepsis (n = 2); Others (n = 6)	**G-ve. Sepsis (n = 7), with 2 blood stream infection**: *P.aeruginosa, E. cloacae, E.Coli, Klebsiella spp, H. influenzae, C. koseri; * ***G+ve. Sepsis (n = 3)*** *: Streptococcus spp, Enterococcus spp, S. Aureus; * ***Mixed G+ve. & G-ve. Blood stream infection (n = 1)*** *: S. aureus; P. aeruginosa*
**Sweden**	**None Sepsis**	All patients were at admission subjected to blood culture, but discharged without evidence of bacterial infection. Mostly viral infections (e.g. influenza, gastro-enteritis) or inflammatory reactions (e.g. reactive arthritis, pseudo-gout)	No relevant positive culture results
	**Sepsis**	Lower respiratory tract infection/Pneumonia (n = 8); Urinary Tract Infection/Pyelo-nephritis (n = 5;) Endocarditis (n = 2); Biliary Sepsis (n = 1); Septic Arthritis (n = 1); Tissue Disease/Abscesses (n = 2); Others (n = 3)	**Blood Stream Infection/Positive blood culture (n = 17)**: *S.aureus (n = 4); E.coli (n = 4); S. pneumoniae (n = 2); Streptococcus spp (n = 4) E.faecalis (n = 1); S. marcescens (n = 1); P. aeruginosa (n = 1)*

23 septic patients and 15 none septic patients recruited in Örebro University Hospital, Sweden were used as an independent validation cohort. Within the sepsis group, 17 had positive blood cultures and the remaining 6 cases were diagnosed clinically. 10 septic patients (43%) were admitted into ICU after blood samples were taken. Clinical diagnoses for both patient groups are listed in Table 3 and Table 4.

**Table 4:** Demographic and clinical information (Swedish Cohort).

	Patients with no evidence of sepsis (n = 15)	Patients with severe sepsis/septic shock (n = 23)	p-value
**Age**	69 (23–75)	68 (33–78)	0.89
**Male**	8 (53.3%)	15 (65.2%)	0.46
**Septic shock**	0	5 (21.7)	—
**ICU admission**	0	10 (43.5%)	—
**1-month mortality rate**	0	5 (21.7%)	—
**CRP**	44(1.3-202)	167 (17-488)	0.001

Data are presented as numbers (percentages) for categorical variables and as median values (ranges) for continuous variables. Statistics: The distribution of sex was tested using Pearson Chi-Square Tests, the rest variables were tested using Mann-Whitney U test.

Septic patients from both cohorts had significantly higher CRP measurements than the SIRS or non-infected group, but no difference in body temperature or neutrophil count was observed in both cohorts despite an elevated white cell count of septic patients in UK cohort (Table 2 and Table 4).

### Identification of microRNA candidates

**Figure 1:**
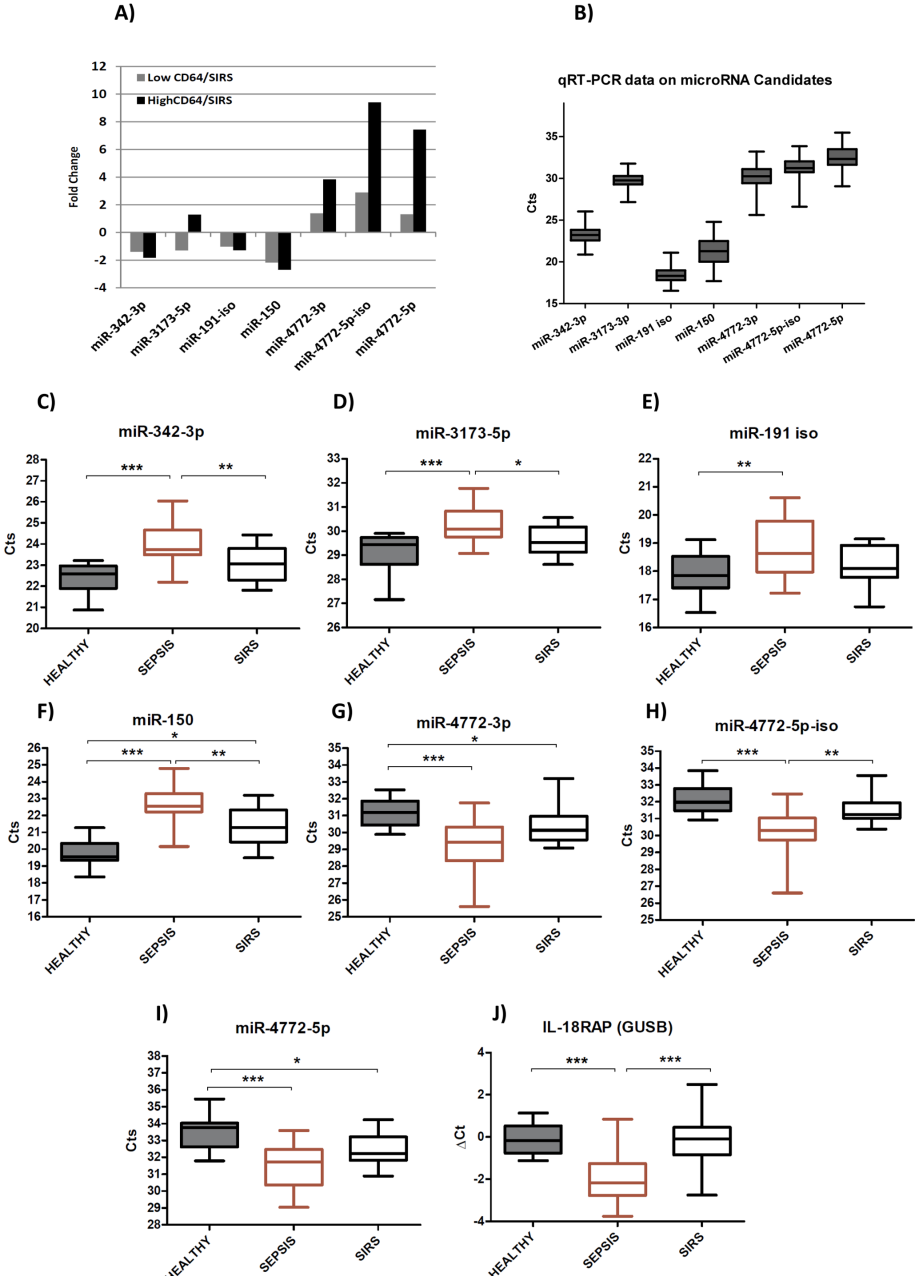
Fold Changes of microRNA sequencing data and candidate microRNA expression validated by qRT-PCR. (**A**) Each bar represents fold increase or decrease in the number of reads for one microRNA candidate comparing sepsis group with SIRS group; grey bar represents fold change data from septic patients with low CD64 expression whereas black bar represents patients with high CD64 expression. A pool of 4 whole blood samples for each group was used for sequencing experiments; (**B**) Box and whisker plot shows microRNA expression in raw Ct values (Y axis) of 7 candidate microRNAs (X axis); n = 61; (**C**) **to** (**I**) microRNA levels of 7 candidates and (**J**) mRNA level of IL-18RAP (normalized to GUSB) in samples from healthy donors (n = 17), sepsis patients (n = 22) and SIRS patients (n = 22) detected by qRT-PCR. Kruskal-Wallis ANOVA test was applied, * significant at p<0.05, ** significant at p<0.01, *** significant at p<0.001.

Pooled RNAs of 4 samples in each groups of healthy volunteers, SIRS or sepsis patients were used for small RNA sequencing. FACS phenotyping showed that neutrophil CD64 expression correlated with sepsis (Figure S1 in File S1). Therefore, sequencing data was grouped as CD64^high^ or CD64^low^ sepsis vs. SIRS (Figure 1 A). Patients were age and sex matched among different groups (Table 5). Out of more than 400 detected miRNA sequences, only those with a fold change ±2 and a number of reads >20 were selected for further analysis (Table 6). The quality of sequencing was validated for a further 3 samples using RNA derived from whole blood, monocyte depleted PBMCs and CD14+ monocytes. 15 to 62 million total reads were achieved with an overall quality of read above 90% (Table S1 in File S1).

**Table 5:** Demographic and clinical information for patients used for sequencing.

	Sepsis patients CD64 high, (n = 4)	Sepsis patients CD64 low, (n = 4)	SIRS patients (n = 4)	*p*-value
**Age**	65 (61–74)	73 (52–87)	70 (43–84)	0.4724
**Male**	3 (75%)	1 (25%)	2 (50%)	0.3679
**Clinical Diagnosis**	Intra-abdominal Sepsis (n = 2; Acute Kidney Infection in both cases); Pneumonia (n = 1); Biliary Sepsis (n = 1)	Pneumonia (n = 2); Lower respiratory tract infection (n = 2)	Coronary Artery Bypass Grafts (n = 1) Aortic Valve Replacement (n = 1); Trans Apical Aortic Valve Replacement (n = 1); Post operative trauma (n = 1, tracheal dilatation)	n/a
**CRP**	328 (203–498)	153 (45–305)	68 (25–100)	0.0559
**WBC count**	20.7 (15.5–23.7)	15.9 (13.3–18.8)	15.2 (12.5–19.5)	0.0775
**Neutrophils count**	19.3 (13.5–22.8)	14.0 (11.2–17.3)	12.7 (9.9–17)	0.0684
**Temperature**	36.0 (35.2–36.7)	36.3 (34.4–37.7)	36.7 (36.5–37.0)	0.3829

Data are presented as numbers (percentages) for categorical variables and as median values (ranges) for continuous variables. Statistics: The distribution of sex was tested using Pearson Chi-Square Tests, the rest variables were tested using Kruskal-Wallis ANOVA test.

**Table 6:** Sequencing information of a selection of candidate microRNAs*.

No	MicroRNA Candidates Information			Reads per Million (RPM)			
	Mirbase name	Sequence	Length (nt)	HEALTHY(n = 4)	SIRS (n = 4)	Sepsis	
						High CD64 (n = 4)	Low CD64 (n = 4)
1	miR-342-3p	TCTCACACAGAAATCGCACCCGT	23	1135.61	91.36	49.96	65.19
2	mir-3173-3p	TGCCCTGCCTGTTTTCTCCTTT	22	19.34	0.48	0.62	0.37
3	miR-191-iso	CAACGGAATCCCAAAAGCAGCT	23	24388.49	2594.81	2010.12	2549.78
4	miR-4772-3p	CCTGCAACTTTGCCTGATCAGA	22	6.02	2.89	11.11	3.99
5	miR-4772-5p-iso	TCTGATCAGGCAAAATTGCAGA	22	0.51	1.21	11.39	3.49
6	miR-4772-5p	TGATCAGGCAAAATTGCAGACT	22	0.13	0.65	4.83	0.85
7	miR-150	TCTCCCAACCCTTGTACCAGTG	22	649.83	151.43	56.08	69.54
8	miR-146a	TGAGAACTGAATTCCATGGGTT	22	273.42	526.72	383.41	485.05
9	miR-146b-5p	TGAGAACTGAATTCCATAGGCT	22	235.67	366.9	296.69	389.04
10	miR-223	TGTCAGTTTGTCAAATACCCCA	22	37072.6	9987.9	16585.9	10708.9
11	miR-125b	TCCCTGAGACCCTAACTTGTGA	22	399.6	28.97	41.77	24.57
12	miR-125a-5p	TCCCTGAGACCCTTTAACCTGTGA	24	138.77	18.06	15.59	17.92

**Figure 2:**
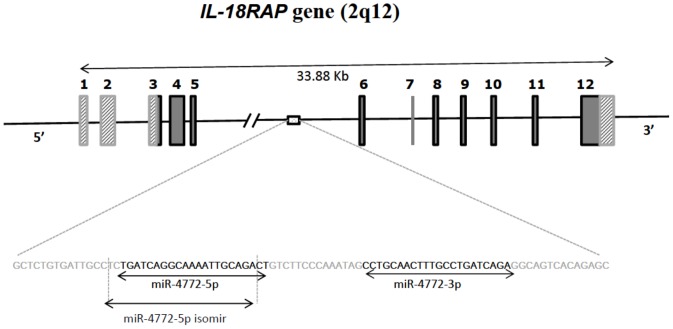
Simplified diagram illustrates the genomic location of miR-4772 family on Chromosome q12.1 within intron 5 of IL-18 RAP.

* Over 400 microRNA were identified in whole blood samples but only ones that of potential diagnostic interest or previously reported related with infection and sepsis were listed;

A group of microRNAs related to miR-4772 demonstrated potential in differentiating sepsis from SIRS, namely miR-4772-3p, miR-4772-5p and miR-4772-5p-iso (an isomir of miR-4772-5p). miR-4772 was the first identified microRNA family upregulated during sepsis, with a distinctive fold change compared with the SIRS group, especially in CD64 high samples (Figure 2 A). MiR-4772-5p-iso drew our particular interest for two reasons; firstly it was more abundantly expressed than mir-4772-5p, the isoform reported first and registered on mirbase; secondly it yielded the largest sepsis/SIRS fold increase among the 3 (average of 6 fold). *Hs-mir-4772* is located in intron 5 of Interleukin 18 receptor accessory protein (IL-18RAP, Figure 2). Given that microRNAs embedded within protein coding genes are often co-regulated, we also assessed IL-18RAP mRNA expression as a potential biomarker of sepsis. miR-150 was chosen as a potential candidate because it decreased markedly during inflammation as previously reported [10] and also show considerable differences in expression between SIRS and Sepsis (Figure 1 A).

We also identified 3 microRNAs, miR-342, miR-3173-5p and miR-191, which were profoundly decreased (>10 fold) during SIRS and sepsis compared with healthy subjects (Table 6). Their dynamics during inflammation has not previously been reported in the literature. Although the fold differences between SIRS and sepsis for these 3 candidates were overall less than to miR-4772 family (Figure 1 A), they were analyzed further because of their potential biological relevance in systemic inflammation.

Candidates showing discriminative potential for sepsis were chosen and validated by qRT-PCR with RNA from 61 whole blood samples (healthy n = 17, sepsis n = 22, SIRS n = 22) (Figure 1 B). Twelve different microRNAs were tested by qRT-PCR in all patient samples. Seven of these were retained because they correlated well with the sequencing data and showed a significant p value (p<0.05) by ANOVA (Table 6, Figure 1). The expression pattern confirmed the sequencing data. Interestingly, miR-4772-5p-iso was expressed at a higher level than miR-4772-5p, but not as highly expressed as miR-4772-3p (Figure 1 B).

Analysis of the qRT-PCR results then focused on whether candidates had differential expression in the sepsis patient group compared with both the SIRS group and healthy controls. All 7 microRNA candidates had highly significantly different expression between septic patients and healthy donors (*p<0.001*) (Figure 1 C–J). First of all, analysis was focused on which candidates distinguish sepsis from both SIRS and healthy subjects. miR-342-3p, miR-3173-5p and miR-4772-5p-iso had significantly increased expression in septic patients compared with the other two groups. The mRNA level of IL18RAP (the gene which hosts miR-4772-5p) normalized by β Glucuronidase, GUSB was also increased in septic patients compared with both SIRS and healthy controls (Figure 1 H). miR-4772-5p-iso was the most significantly up-regulated microRNA in the septic group (*p<0.001*). Furthermore, miR-150 not only distinguished sepsis from SIRS and healthy subjects, but also showed an elevated level in SIRS compared with healthy subjects (Figure 1 F). On the other hand, miR-4772-3p and miR-4772-5p were increased both in sepsis and SIRS condition compared with healthy subjects (Figure 1 G&I), which might be more related with inflammation rather than infection. A direct correlation between IL18 levels and miR-150 down-expression has previously been shown (G.calin). As IL18 and IL18RAP are closely located, we hypothetize that IL18RAP and its' intronic microRNA miR-4772-5p are co-ordinately regulated.

### LDA modeling generates a novel microRNA score that distinguishes septic patients from SIRS

Further analysis employed a multivariate ANOVA test to select the best microRNA candidates in order to construct a scoring system in distinguishing sepsis from SIRS. Based on a multivariate test ranking, miR-150 and miR-4772-5p-iso were the top two candidates and therefore chosen for a Linear Discriminant Analysis (LDA) to generate a “sepsis score” (Table 7).

**Table 7:** Multivariate ANOVA analysis of UK cohort (Sepsis vs. SIRS).

Gene	Df	Sum Sq	Mean Sq	F value	Pr(>F)	Significance
miR-150	1	2.533136771	2.533136771	15.70689721	**0.000404617**	***
miR-4772-5p-iso	1	2.068628272	2.068628272	12.82667877	**0.001151121**	**
miR-191	1	0.424406741	0.424406741	2.631564603	0.114886364	
miR-342-3p	1	0.063767783	0.063767783	0.395396737	0.534082531	
miR-4772-3p	1	0.048997072	0.048997072	0.303809879	0.585454953	
miR-3173-5p	1	0.001356836	0.001356836	0.008413161	0.927508062	
IL18RAP (noramlized to GUSB)	1	0.000382344	0.000382344	0.002370752	0.961478409	

* significant at p<0.05,

** significant at p<0.01,

*** significant at p<0.001.

**Figure 3:**
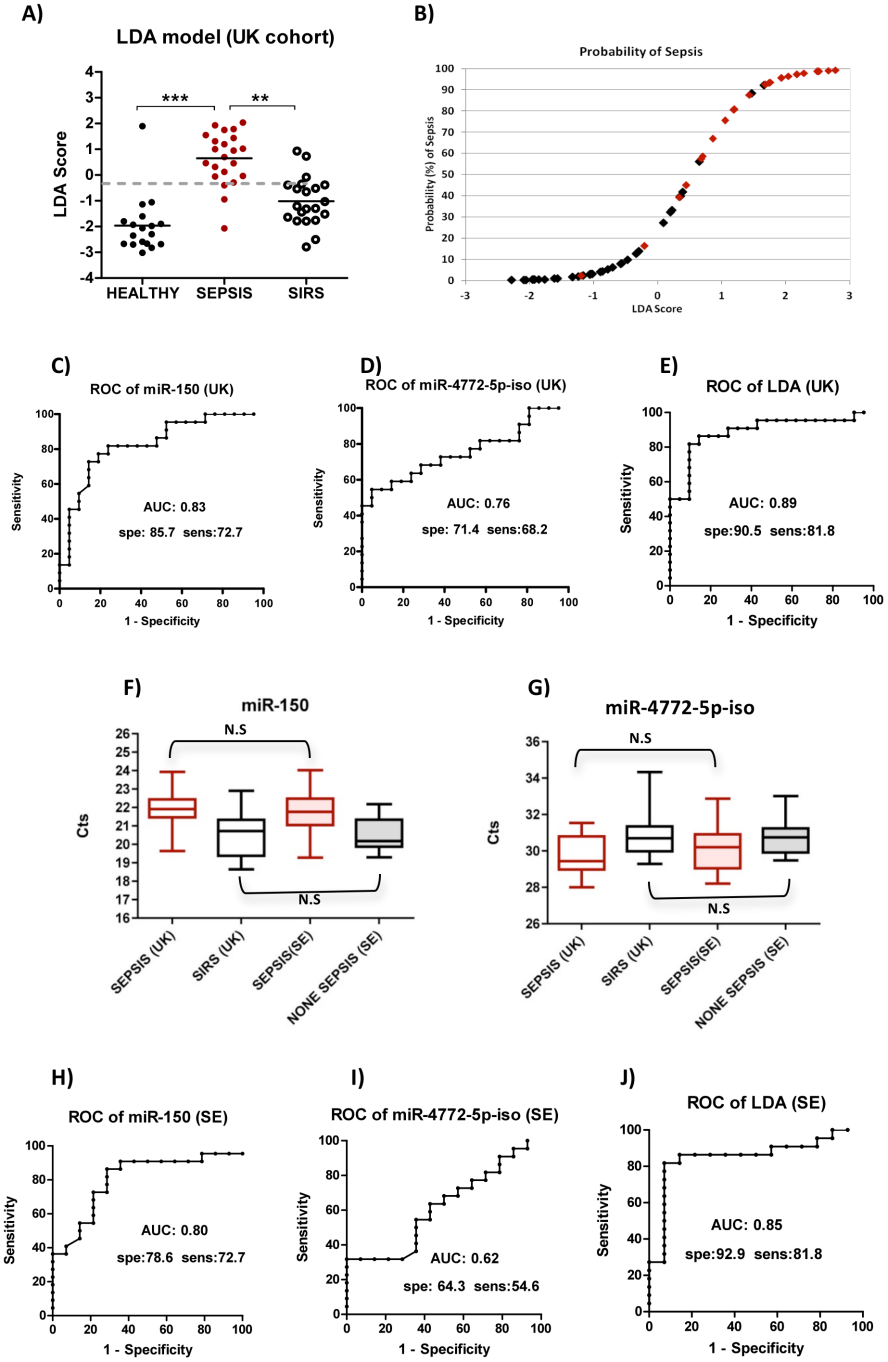
Construction and Validation of a Linear Discriminant Model for diagnosing sepsis from SIRS. (**A**) LDA score was achieved using linear discriminant analysis (LDA) based on results of miR-4772-5p-iso and miR-150 in UK cohort; Mann-Whitney U test were applied. *** significant at p<0.001; (**B**) Prediction plot based on LDA score (X axis) and re probability of sepsis (Y axis), Red dots: Sepsis, Black dots: SIRS; ROC Curves demonstrate the diagnostic capacities of (**C**) miR-150 alone, (**D**) miR-4772-5p-iso alone and (**E**) LDA score; (**F**) miR-150 expression and (**G**) miR-4772-5p-iso expression of 2 patient groups from both UK and Sweden, Kruskal-Wallis ANOVA test was applied; ROC curves show the diagnostic power of (**H**) miR-150 and (**I**) miR-4772-5p-iso alone; (**J**) LDA score from Swedish cohort.

The LDA score discriminated sepsis from both SIRS and healthy groups at a highly significant level in the training cohort (Figure 3 A). When a cut-off value of −0.2 was chosen, sepsis was successfully diagnosed in 90.9% of patients (20/22, Figure 3 B). LDA achieved an AUC of 0.90, with better specificity (90.5%) and sensitivity (81.8%) than miR-150 or miR-4772-5p-iso alone, which only gave specificities and sentisitivities of 85.7% and 72.7%, or 71.4% and 68.2% respectively (Figure 3 C–E).

### Validation of the LDA score in a second independent cohort

qRT-PCR experiments were performed for miR-150 and miR-4772-5p-iso and results for both sepsis and control groups from Sweden were not statistically difference to the equivalent UK groups (Figure 3 F&G). When the LDA score was constructed using the same algorithm, the diagnostic power increased to an AUC of 0.85 while miR-150 alone had an AUC of 0.80 and miR-4772-5p-iso had 0.62 (Figure 3 H–J). 81.8% (18/22) sepsis cases were successfully predicted using LDA score with only a 7.1% (1/14) false positive rate. LDA scores from the two cohorts generated an overall diagnostic accuracy of 86%.

### Differential expression of microRNAs is maximal in the monocyte fraction from peripheral blood

**Figure 4:**
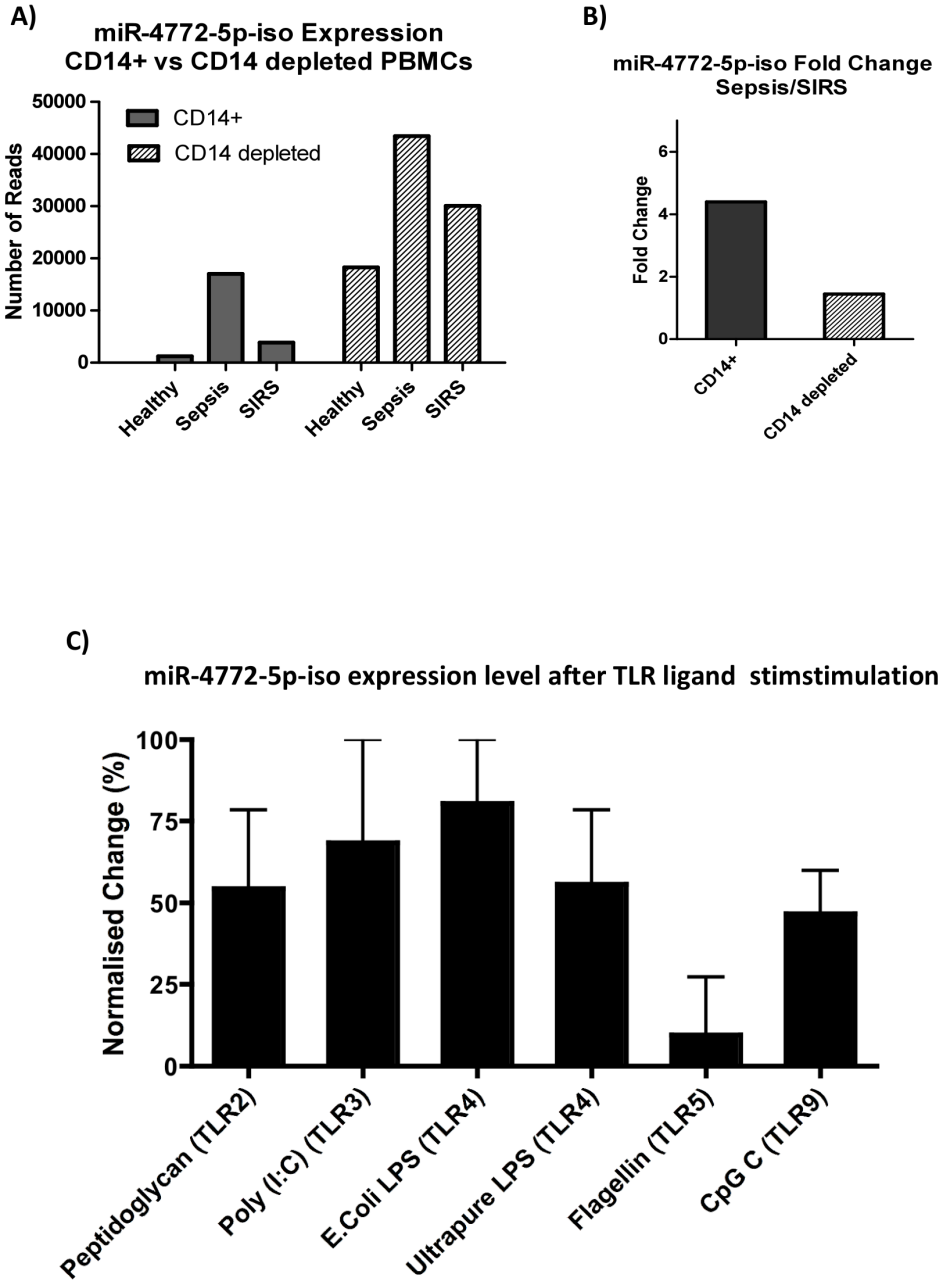
Expression of miR-4772-5p-iso in CD14+ monocytes between sepsis and SIRS compared with CD14 depleted PBMCs, and expression levels in healthy monocytes stimulated by TLR ligands. (**A**) Small RNA sequencing of CD14+ monocytes vs CD14 depleted PBMCs for miR-4772-5p-iso expression in Healthy, Sepsis, and SIRS samples (experiments were performed using RNA from a pool of samples in each group, see Table S1.). (**B**) Fold changes of sequencing results based on the number of reads/million in Sepsis vs SIRS group; (**C**) **qRT-PCR results of** microRNA miR-4772-5p-iso expression in CD14+ monocytes from healthy donors stimulated with different TLR ligands for 24 hours; does and source of TLR ligands listed in methods section. qRT-PCR was done using triplicates. Fold changes were calculated based on RPMI medium alone (see method) then normalised to percentage of maximum effect in each experiment, error bars represents Standard Error of the Mean (SEM); n = 2.

To study which cell subset was more likely to contribute to the up-regulation of miR-4772-5p-iso in sepsis patients, peripheral blood mononuclear cells (PBMC) from healthy controls, sepsis patients and SIRS patients were isolated and further purified into CD14+ monocytes and CD14 depleted PBMCs (>90% CD3+ T cells). Small RNA sequencing of these two fractions revealed that, although miR-4772-5p-iso is expressed at high abundance in CD14 depleted PBMCs (Figure 4 A), expression in CD14+ monocytes generated more than a 4 fold change between sepsis and SIRS patient groups whereas only less than 2 fold change was observed in CD14 depleted population (Figure 4 B). These data suggest that the differential microRNA signal came mainly from the circulating monocyte population.

### Toll-like receptor (TLR) ligand stimulation up-regulates miR-4772-5p-iso expression in primary human monocytes

As sepsis is driven by microbial signals via TLRs, we sought to address the mechanistic hypothesis that TLR ligation would cause the observed microRNA expression changes. Purified primary human monocytes from healthy volunteers were stimulated by a broad panel of TLR ligands and the expression of miR-4772-5p-iso was measured after 8 and 24 hours of stimulation. The experiment was repeated twice and for each experiments qRT-PCR was done in triplicates. Data were then normalised to the percentage of maximum effect of each experiment. There were no significant changes in expression after 8 hours of culture (data not shown), but after 24 hours there were significantly increase in the expression of miR-4772-5p-iso (Figure 4 C) to the majority of TLR ligands compared to baseline. Primary human monocytes do not survive well for longer than 24 hours *in vitro* following TLR ligands challenge at given does, so later time points were not tested.

## Discussion

This study aimed to identify novel biomarkers to rapidly diagnose sepsis in ICU. Through small RNA sequencing of whole blood samples, novel and known microRNAs were identified that were differentially expressed in septic patients and controls. This finding was further confirmed by qRT-PCR and a composite signature of miR-150 and miR-4772-5p-iso was generated by an LDA model which possesses 90.5% specificity and 81.8% sensitivity in distinguishing sepsis from SIRS. This novel signature was then validated in an independent cohort and the results in the two cohorts showed an 86% diagnostic accuracy for sepsis. *In vitro* work revealed that miR-4772-5p-iso was upregulated in primary peripheral blood monocytes after a 24 h challenge with specific TLR ligands, providing a potential mechanistic explanation for the observed data.

Research focusing on more accurate and rapid diagnosis of sepsis has highlighted methods that include specific serum and cell surface proteins and bacterial DNA detection [25], [26]. Neutrophil CD64 expression was first reported to be increased from patients with acute bacterial infection compared with healthy controls over 20 years ago [27]. A recent meta-analysis of 14 publications summarized that the average sensitivity and specificity of neutrophil CD64 was 79% and 91% respectively [28], which was reproduced in our study setting. We also found neutrophil CD64 expression correlated with the APACHE II score, with indicates the severity of sepsis (Figure S1 in File S1). However, due to the complexity of using FACS analysis at the patients' bedside, CD64 has not been adopted into routine clinical practice.

A large body of research has shown that microRNAs control important processes such as cell proliferation, adhesion, apoptosis and angiogenesis (reviewed in [29], [30], [31]). Recent publications also demonstrate that microRNAs can be considered as diagnostic markers [8], [9], [10], [32], [33], [34]. The recent development of small RNA deep sequencing has revolutionised microRNA identification, especially those with potential diagnostic or prognostic value [35], [36], [37], [38], [39].

It has been suggested that decreased plasma miR-150 is a diagnostic and prognostic marker for sepsis [10]. It was also reported that serum miR-146a and miR-223 levels were lower in septic patients compared with SIRS, generating an AUC of 0.858 and 0.804 respectively [14]. Wang *et al* studied a cohort of 214 sepsis patients and found that a combination of 4 microRNA markers in serum (miR-15a, miR-16, miR-193* and miR-483-5p) and sepsis clinical scores predicted 28 days survival rate with a sensitivity of 88.5% and a specificity of 90.4% [33]. We employ massively parallel high throughput small RNA sequencing technique to identify candidates from the entire microRNAome that are differentially expressed in whole blood from patients with sepsis and SIRS. This putative “sepsis signature” was then validated using real time PCR in a clinical study. Our findings confirm that miR-150 is down regulated during sepsis compared with SIRS and healthy subjects and yielded a comparable AUC of 0.83 [10]. miR-146a and miR-223 were also detected in our sequencing assays, but did not generate a significant fold change compared to the other candidates (Figure S2 in File S1). Of note,our experiments were done using RNAs extracted from whole blood samples, which contain intracellular microRNAs that might have contributed to the difference compared to other published studies. However by using whole blood we were able to perform deep sequencing, which allowed a novel microRNA to be discovered. We discarded microRNAs largely contributed by red cells or platelets (e.g miR-451). We decided to use whole blood because current extraction methods for serum tend to bias microRNA expression [40].

Two novel microRNAs, namely miR-342-3p and miR-3173-5p were also decreased significantly in septic patients compared to SIRS. Interestingly, the microRNA miR-4772 family was found to be the only one significantly up-regulated in the sepsis group compared to healthy subjects. Particular attention was paid to miR-4772-5p iso, which was significantly upregulated compared with both healthy subjects and SIRS patients.

Using Linear Discriminant Analysis, choosing a pool of SIRS and healthy subjects as a control, we successfully constructed a statistical model using real-time PCR data obtained from a UK training cohort, which demonstrated miR-150 and miR-4772-5p-iso to be the best two candidates to diagnose sepsis. This result was cross-validated with samples from an independent cohort in Sweden of patients with severe sepsis or septic shock. Patients in the validation cohort were selected retrospectively from a total of 1093 patients based on detailed clinical information, lab results and clinical outcome [19]. Compared to the ICU environment, our second cohort gave a clearer defined diagnosis which provided more confidence in evaluating the accuracy of our proposed biomarkers.

Our unpublished data from more than 300 sequencing experiments in different tissues, such as embryonic cells and various tumors, show that miR-4772-5p-iso is mainly expressed in monocytes and T cells, indicating the specificity of this microRNA for the immune system. This is the only candidate among over 400 microRNAs from the sequencing data that is upregulated. miR-4772-5p-iso has no murine homologue and it is plausible that species-specific microRNAs are likely to represent more specific biomarkers for human disease. These data indicate that miR-4772-5p-iso may also play an important biological function during sepsis. miR-4772-5p-iso is located in an intronic region of IL-18RAP (Figure 2), which was also increased during sepsis. IL-18 itself has previously been reported to be upregulated during sepsis [10], [41] and given its genomic location adjacent to IL18RAP, is likely to be co-ordinately regulated.

To determine which blood cell subpopulation was responsible for the observed microRNA expression changes, we purified CD14+ monocytes and monocyte-depleted peripheral blood mononuclear cells (PBMC) from healthy donors. Sequencing data suggested that although CD14 depleted cells (which are mainly T lymphocytes) expressed high levels of miR-4772-5p-iso, CD14+ monocytes generated a much better discriminative signal between sepsis and SIRS. This finding indicated that miR-4772-5p-iso may be functionally more related to innate rather than adaptive immunity especially in a situation such as sepsis. Incubate of monocytes with TLR ligands showed that the majority of TLR ligands upregulated this particular microRNA. Given that sepsis is associated with TLR ligation by exogenous microbial ligands, these data support the hypothesis that upregulation of this specific microRNA may be a useful mechanistic biomarker and a sensitive way of detecting TLR ligation in circulating blood monocytes.

In summary, we have identified a microRNA based molecular signature that reliably discriminates sepsis from SIRS. Given that this test can be performed rapidly at the point of care, it has the potential to transform the management of this severe human disease. Despite the limited sample size in the current study, we have been able to replicate that initial finding in a separate cohort. Given the complex nature of sepsis/SIRS, randomised prospective large scale clinical trials are now needed to determine the value of this potential microRNA based biomarker in larger patient groups.

## Supporting Information

File S1 **File S1 contains 4 parts: supporting information S1, Figure S1, Table S1 and Figure S2.** Figure S1. Neutrophil CD64 expression in different groups. (A) Each point represents the expression of CD64 on neutrophils for an individual patient expressed as MFI (Mean Fluorescent Intensity). The bar represents the geometric mean of each group. Kruskal-Wallis ANOVA test was applied. *** significant at p<0.001. (B) A representative FACS dot plot showing neutrophils gated by CD66b and high side scatter. (C)&(D) show histogram of isotype FITC expression and CD64 expression of two ICU patients representing high (black line) or low (grey solid) expression of CD64. (E) ROC Curves demonstrate the diagnostic utility of CD64 in comparison with WBC, CRP and temperature for sepsis in ICU. (F) The correlations of neutrophil CD64 with APACHE II score; (G): The correlations of neutrophil CD64 with SOFA score. Figure S2. miR-146a and miR-223 expression level in different groups (UK cohort). Each bar represents the expression of miR-146a (left) or miR-223 (right) in healthy volunteers, Sepsis patients or SIRS patients; Kruskal-Wallis ANOVA test was applied. *** significant at p<0.01; *** significant at p<0.001.
(DOC)
