# Supplementary material for: Genome-Wide Sequencing of Cellular microRNAs Identifies a Combinatorial Expression Signature Diagnostic of Sepsis
Source: PLoS One. 2013 Oct 16;8(10):e75918. doi: 10.1371/journal.pone.0075918 (PMC3797812; doi:10.1371/journal.pone.0075918)
Supplement: File S1 — contains 4 parts: supporting information S1, Figure S1, Table S1 and Figure S2. Figure S1. Neutrophil CD64 expression in different groups. (A) Each point represents the expression of CD64 on neutrophils for an individual patient expressed as MFI (Mean Fluorescent Intensity). The bar represents the geometric mean of each group. Kruskal-Wallis ANOVA test was applied. *** significant at p<0.001. (B) A representative FACS dot plot showing neutrophils gated by CD66b and high side scatter. (C)&(D) show histogram of isotype FITC expression and CD64 expression of two ICU patients representing high (black line) or low (grey solid) expression of CD64. (E) ROC Curves demonstrate the diagnostic utility of CD64 in comparison with WBC, CRP and temperature for sepsis in ICU. (F) The correlations of neutrophil CD64 with APACHE II score; (G): The correlations of neutrophil CD64 with SOFA score. Figure S2. miR-146a and miR-223 expression level in different groups (UK cohort). Each bar represents the expression of miR-146a (left) or miR-223 (right) in healthy volunteers, Sepsis patients or SIRS patients; Kruskal-Wallis ANOVA test was applied. *** significant at p<0.01; *** significant at p<0.001. (DOC) [file pone.0075918.s001.doc]

**Supporting Information S1. Inclusion and Exclusion Criteria:**

There were 3 groups included in the study: healthy volunteers, SIRS patients without bacterial infection and septic patients. Diagnosis of a patient as septic required two or more of SIRS criteria with a clinically suspected source of infection at the time of recruitment. The SIRS group required two or more of the SIRS manifestations with no suspected source of infection at the time of recruitment. The judgment of presence or absence of a source infection was made by the research nurses in discussion with the attending physician.

Exclusion criteria were < 18 years old, pregnancy and more than 48h since the first sign of inflammation. 20ml whole blood was taken for each subject after assent had been obtained from the next of kin. Samples were stored at 4°C while waiting for collecting by the researcher.


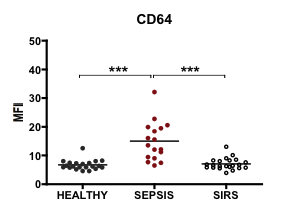

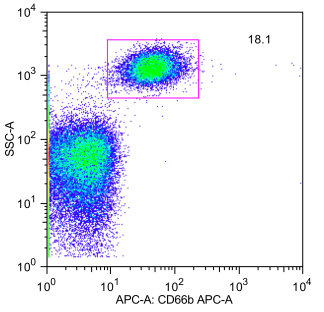

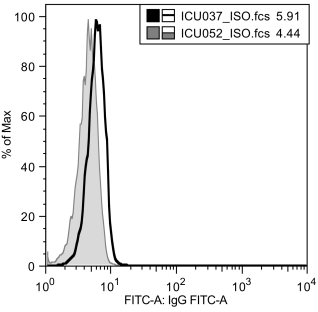

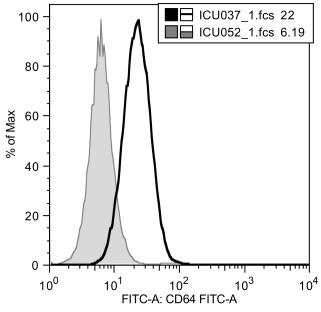


**A B C D**

**E F G**


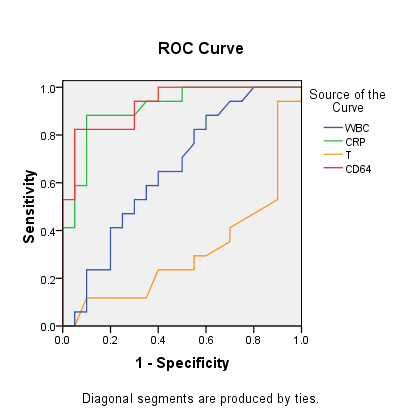

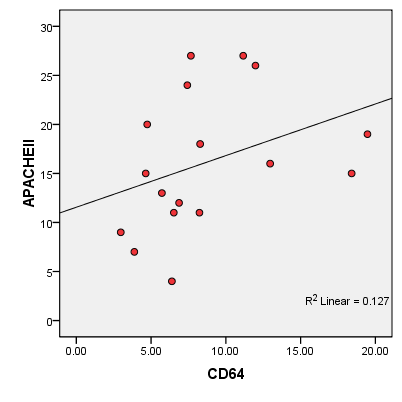

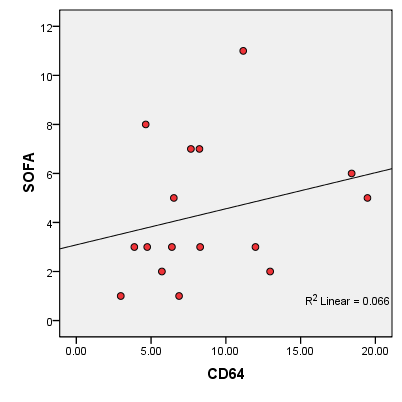


**Figure S1. Neutrophil CD64 expression in different groups**

Table S1. Sequencing Validation using RNA from whole blood, monocytes delpeted PBMCs

and CD14+ monocytes

| **Group** | **Tissue/Cell** | **Samples** | **Number of reads** | **Number of reads mapped to the human genome(HG19)** | **Quality of the reads (phred score > Q30)** |
| --- | --- | --- | --- | --- | --- |
| Sepsis | Whole Blood | Pool of 4 patients | 62 747 412 | 56 925 855 | 90.60% |
| Sepsis |  | Pool of 4 patients | 44 575 309 | 41 325 390 | 90.80% |
| Healthy |  | Pool of 4 patients | 40 593 107 | 37 726 241 | 91.10% |
| Sirs |  | Pool of 4 patients | 49 964 795 | 46 449 781 | 91.00% |
| Healthy | CD14 depleted PBMC | Pool of 4 patients | 15,667,381 | 11,617,218 | 89.17% |
| Sepsis |  | pool of 5 patients | 35,818,967 | 30,309,961 | 92.07% |
| Sirs |  | pool of 5patients | 34,079,526 | 30,784,742 | 91.40% |
| Healthy | CD14+ Monocytes | Pool of 7 patients | 23,187,363 | 8,054,227 | 93.61% |
| Sepsis |  | Pool of 5 patients | 26,730,224 | 13,337,444 | 93.46% |
| Sirs |  | Pool of 3 patients | 32,781,564 | 27,622,241 | 94.29% |

**Figure S2. miR-146a and miR-223 expression level in different groups (UK cohort)**
